# Feasibility and acceptability of a physical activity intervention to reduce prenatal cannabis use: results of an open pilot trial

**DOI:** 10.3389/fpsyt.2026.1729092

**Published:** 2026-01-30

**Authors:** Cynthia L. Battle, Sarah E. Dreyer-Oren, Andrea Vijil Morin, Morgan N. Hoyt, Jane Metrik, Ana M Abrantes

**Affiliations:** 1 Warren Alpert Medical School, Brown University, Providence, RI, United States; 2 Butler Hospital, Providence, RI, United States; 3 Women & Infants Hospital of Rhode Island, Providence, RI, United States; 4 Center for Alcohol and Addiction Studies, Brown University School of Public Health, Providence, RI, United States; 5 Providence VA Medical Center, Providence, RI, United States

**Keywords:** anxiety, cannabis (marijuana), depression, intervention - behavioral, physical activity, pregnancy

## Abstract

Cannabis is commonly used among reproductive-aged individuals, and prenatal cannabis use (PCU) has increased dramatically in recent years, despite guidance warning of possible adverse outcomes. Physical activity interventions have been shown to reduce substance use in other populations. Building on this, we examined the feasibility and acceptability of a 10-week prenatal walking intervention in a small trial with 16 pregnant individuals who were seeking to reduce PCU. Participants wore a Fitbit to track activity and attended 6 sessions designed to promote gradual increases in daily step count. Indicators of feasibility, acceptability and safety were assessed, as were changes in cannabis use, physical activity, depression and anxiety. Results suggest the intervention was feasible and acceptable; most women (88%) completed the intervention, attending on average 5.8 of 6 sessions, with strong compliance to Fitbit wear. No adverse events were reported. Findings provide preliminary evidence for intervention efficacy: 62.5 percent of participants endorsed PCU at baseline vs. 16.6% by the 36 week assessment; in addition, by endpoint, physical activity increased from an average daily stepcount of 5738 at baseline to 6562, and anxiety and depression were significantly lower. Participants reported high satisfaction with the intervention on a satisfaction survey and in an interview. Participants voiced appreciation for the accountability provided by the intervention, and support for making gradual, achievable changes in behavior. Though preliminary, findings suggest a physical activity intervention could be a valuable strategy to help reduce PCU. Further research is needed to evaluate the intervention in a more rigorous controlled trial.

## Introduction

Cannabis is the drug most frequently used in pregnancy (1–3), and rates of prenatal cannabis use (PCU) are rising, having increased from 5.5% of pregnancies in 2012 to 9.0% of pregnancies in 2022 (4). The increase in PCU contrasts with decreasing rates of prenatal alcohol and tobacco use during the same period (1). Of those who use cannabis before pregnancy, at least 25% to 41% will continue to use once pregnant (5–7); further, many individuals who quit cannabis while pregnant will return to use postpartum (8). A substantial proportion of those who engage in PCU are using at heavy or problematic levels: 16% of pregnant individuals used daily or near-daily, and 18% meet diagnostic criteria for cannabis use disorder (9).

The American College of Obstetricians and Gynecologists (10), American Academy of Pediatrics, and other public health organizations caution against PCU, citing harms for both pregnant people and their infants (10–13). Maternal health issues associated with PCU include gestational hypertension, pre-eclampsia, eclampsia, gestational diabetes, gaining too much or too little weight, and anemia (14). Infants exposed to cannabis prenatally have greater risk for pre-term birth, being small size for gestational age, and perinatal mortality (15). Though there are conflicting reports, some studies have found longer-term developmental problems such as increased risk of autism spectrum disorder and behavioral and attention problems (16, 17).

In the general population, cannabis use can be motivated by desire to cope with negative emotions (18), to enhance social interactions, to relax, or to use for medicinal reasons (e.g., for pain, anxiety (19, 20)). Those who use cannabis prenatally may share these motivations, and also seek to relieve pregnancy symptoms such as nausea, appetite problems, mood problems, anxiety, pain, and sleep disturbance (6, 21–26). For some, cannabis use may persist during pregnancy not only due to potential for mitigating pregnancy symptoms, but also due to lack of guidance about potential risks associated with PCU from prenatal care providers. Although many providers caution patients about risks of PCU, research suggests that a substantial proportion may not provide any guidance about use or mention risks (27–29). This breakdown in patient-provider communication may relate to providers’ lack of training, or not having sufficient treatment options to provide those wishing to reduce use (30, 31). Some patients may also have reservations about disclosing use to providers (32) due to either stigma (33) or fear of child protective services involvement (34).

Despite often-conflicting messages regarding dangers of PCU, many pregnant people who use cannabis are cognizant that PCU confers some risk (35), and often attempt to reduce or abstain if pregnant (7, 36). Some interventions have been designed to reduce prenatal substance use in general, yet few have been designed for cannabis use specifically. A systematic review identified nine trials targeting reduction of PCU, primarily utilizing cognitive behavioral therapy (CBT), motivational interviewing (MI), motivational enhancement therapy (MET), and psychoeducation (37). While the authors concluded that there may be promise to the interventions studied, they noted most trials were non-randomized and/or underpowered. In addition, designs often included a mix of individuals using cannabis and other substances making results difficult to interpret. Authors urged for more research on interventions targeting PCU specifically, noting that the brief (often single-session) interventions studied to date may not be sufficiently intensive to support cessation or a meaningful reduction in use. They also noted that scaling interventions such as CBT or MET may be challenging as they rely upon trained providers. Last, PCU interventions to date have focused on pregnant cannabis users broadly, not on those with mental health symptoms, who are significantly more likely to continue cannabis use prenatally (37). To our knowledge, no trials have been conducted with this at-risk group.

Evidence suggests that engaging in regular physical activity may help people reduce substance use behaviors. Specifically, meta-analytic studies indicate physical activity can reduce withdrawal symptoms, cravings, anxiety, stress, depression, and in some cases, substance use disorder symptoms (38–40). Similarly, in our work, we found that a lifestyle physical activity intervention – that is, increasing daily physical activities (e.g., intentionally taking stairs rather than an elevator, parking further way from the door of a building, building in time for brief walks) improved abstinence outcomes, depression, anxiety, and stress among depressed women in early recovery from alcohol use disorder (41, 42).

A lifestyle physical activity may be an especially apt intervention for PCU. Engaging in regular physical activity is encouraged as a strategy to promote better health during pregnancy, by helping individuals maintain cardiovascular and lung capacity, improve energy levels, and reduce prenatal health risks such as gestational diabetes, pre-eclampsia, and digestive problems (43–45). Further, physical activity can help ameliorate pregnancy symptoms such as pain and appetite disturbance –symptoms that have been shown to prompt PCU (6, 21–24). Physical activity may also help people manage cravings, and provide a healthy, distracting alternative to cannabis use. Although many studies are preliminary in nature, physical activity trials to date have shown promise as a strategy to improve mood among pregnant individuals (46), and improve rates of abstinence from alcohol (in nonpregnant populations) (42); however, to date, there have been no studies examining physical activity as an intervention for PCU.

The current trial, funded by the National Institutes of Health (R34DA055317), was designed to preliminarily evaluate Women Out Walking (WOW), 10-week lifestyle physical activity intervention for pregnant people experiencing elevated symptoms of depression or anxiety, who are seeking to reduce or abstain from PCU. Given the early stage of research, and drawing upon a stage model of treatment development (47) our primary goal was to evaluate the intervention’s feasibility, safety and acceptability. We also examined the preliminary efficacy of the intervention in terms of reducing PCU, increasing physical activity levels, and lowering depression and anxiety.

## Method

### Participants

Pregnant individuals were recruited through outreach to local OB/Gyn clinics in Rhode Island and web advertisements reaching the surrounding area and in neighboring states. Participants were eligible if they were 18+ years of age, had healthy, singleton pregnancies and were medically cleared to exercise by their prenatal provider, were at 12–25 weeks gestation at baseline, had at least minimally elevated symptoms of depression (EPDS >= 7) and/or anxiety (GAD >=5), as measured by the Edinburgh Postnatal Depression Scale (EPDS (48)) or General Anxiety Disorder-7 scale (GAD-7 (49)), for the past 3 months has engaged in <150 minutes/week of moderate intensity physical activity, defined as aerobic activity that requires a moderate amount of effort and noticeably increases heart rate and breathing (50), used cannabis at least once per week in the 3 months prior to pregnancy, and reported a desire to reduce or abstain from PCU. Individuals were excluded if they had a moderate or severe substance use disorder other than cannabis use disorder or nicotine use disorder, used any drugs other than cannabis, nicotine, or alcohol in the past 3 months, had current psychosis, anorexia or bulimia nervosa, cognitive impairment, recent or current suicidality or homicidality, or medical problems that would preclude safe participation, or if they planned to relocate geographically during the study period.

### Procedures

Study activities were approved by the hospital’s institutional review board and the trial was registered with ClinicalTrials.gov on 7-19-22 (#NCT05528380). After a phone screen to determine preliminary eligibility, participants completed an in-person baseline visit. At baseline, following an informed consent process, participants completed self-reports, structured clinical interviews, a urine toxicology screen, and filled out release of information forms allowing providers to communicate with the study team regarding medical clearance to participate.

After enrollment, participants completed an initial monitoring period during which time they wore a wrist-worn activity tracker (Fitbit Inspire) for one week. Participants were instructed to wear the device for 10+ hours daily and not alter activity during this monitoring period. Once cleared, participants were scheduled to begin the intervention. They had the option to complete sessions at the hospital or via remotely. In addition, participants completed assessments at Weeks 3 and 6 after starting the intervention, at end of treatment (EOT; week 10), later in the pregnancy at 36 weeks gestation, and at a 1-month postpartum assessment. To increase retention, participants were provided with compensation for assessments of $10–50 depending on length; intervention sessions were not compensated.

### Physical activity intervention

The Women Out Walking intervention involved use of a Fitbit monitor and brief biweekly sessions, as detailed below.

#### Activity monitor

Participants were given a Fitbit Inspire 3 to wear daily during the intervention and through the postnatal follow-up. Study-generated Fitbit accounts were created, and participants gave permission for investigators to access the Fitbit data throughout the monitoring period. When participants were given the Fitbit at baseline, staff guided them through downloading the Fitbit app on their phone, viewing activity, changing settings, and charging the device.

#### Biweekly sessions

Participants met a study clinician for six, 30-minute sessions over 10 weeks, focused on gently increasing physical activity. Participants were given the intervention manual, which included information on benefits of increasing physical activity, strategies to increase activity, and safety information tailored to safely increasing physical activity during pregnancy. At the first session, (occurring at the Week 0 timepoint) participants and study clinicians identified an initial step count goal that was approximately 500 steps per day greater than the average daily steps they achieved in the prior week, with a maximum of 4500 steps per day at the outset. (E.g., if a participant walked 3900 steps per day on average in the initial monitoring period, they would be assigned 4400 steps for the first week.) In subsequent sessions, which occurred at regular intervals every 2 weeks (Weeks 2, 4, 6, 8 and 10), participants and clinicians collaboratively decided on step count goals based on how participants felt about the goal since the prior session. Generally, participants were guided to increase daily step count goals by no more than 500 steps each week. Given the known benefits of acute, brief bouts of physical activity on mood improvements and reduced craving (51, 52), participants were encouraged to strategically utilize bouts of physical activity (even 5–10 minutes) “in-the-moment” to help manage negative affect or cannabis cravings.

During each session, clinicians completed a health check-in, reviewed participants’ physical activity in the two weeks, inquired about factors that helped or hindered physical activity, and whether the participant used physical activity to cope with emotions or cravings to use cannabis. Each session consisted of brief psychoeducational topic focused on increasing or maintaining walking throughout pregnancy. **Table 1** details the session-by-session content of the intervention.

**Table 1:** Women out walking session content.

Session topic	Description
1: Orientation	Participants are introduced to the physical and mental health benefits of walking during pregnancy and the idea of increasing and maintaining physical activity through tracking step count goals. Participants are given safety guidelines on safe walking during pregnancy.
2: Physical Activity & Mental Health	Discussion of the connection between physical activity and mental health symptoms, general mood, and substance use. Introduction of the concept of using physical activity “in-the-moment” to cope with negative emotions and cannabis cravings. Discussion of ways to incorporate physical activity, including long and short bouts of activity and activities of daily living.
3: Getting Motivated and Staying Motivated	Participants identify reasons they want to prioritize physical activity, and discuss ways to increase external motivation (e.g., rewarding oneself) and internal motivation (e.g., walking with others).
4: Goal Setting and Barriers	Overview of strategies for effective goal setting, including setting short-term goals, making sure goals are specific, measurable, and realistic, and planning and monitoring goals. Includes discussion of getting support with goals. Participants identify internal barriers (e.g., depressive symptoms) and external barriers (e.g., weather) that get in the way of physical activity, and learn strategies on cognitive and behavioral strategies to overcome these barriers.
5: Time Management & Getting Back on Track	Participants learn how to prioritize physical activity in their weekly schedule. Participants also are introduced on how to get back on track after interruptions to exercise, including problem solving strategies and avoiding dichotomous thinking.
6: Maintaining Activity During Pregnancy & After Baby Arrives	Discussion of how to maintain physical activity throughout pregnancy, including throughout the later stages of pregnancy and after baby is born. Includes review of ways to resume physical activity in the context of newborn care (e.g., childcare, using a stroller or baby carrier).

### Measures

#### Feasibility

To examine feasibility, we calculated the average number of sessions completed and adherence to regularly wearing the Fitbit tracker. We also examined the extent to which eligible women decided to enroll in the intervention, and whether providers were willing to respond to requests for medical clearance determinations so their patients could participate.

#### Safety

The Systematic Assessment of Treatment-Emergent Adverse Events (SAFTEE (53)) was used to assess for any adverse and serious adverse events, noting incident severity and relatedness to study participation. Participant self-report and birth record review were also used to assess infant outcomes, such as gestational age at delivery, birth weight, and NICU admission.

#### Acceptability

At the End of Treatment (EOT) Assessment, participants completed the Client Satisfaction Questionnaire-8 (CSQ-8 (54)), an 8-item questionnaire assessing satisfaction with the intervention. Questions are rated on a 4-point scale, from 1 (low) to 4 (high) satisfaction. Also, at EOT, participants completed an exit interview about aspects of the intervention that were helpful or could be improved, and whether they believed it helped them to abstain from PCU.

#### Cannabis use

The Timeline Follow-Back (TLFB) (55) was used to assess cannabis use at baseline and subsequent follow ups. The TLFB is a frequently-used method of substance use assessment that uses a calendar to capture frequency and quantity of use. The initial assessment assessed the period of 90 days prior to baseline; future administrations of the TLFB assessed use since the last assessment. We calculated percent days of cannabis use after learning about pregnancy, as well as percent days used at in the periods leading up to each assessment (Week 3, Week 6, Week 10, 36-weeks gestation, and at 1-month postpartum). We also captured a binary use variable of any use at each timepoint, coded as “0” for no use and “1” for any use. Urine toxicology screens were conducted to assess for cannabis use at in person assessment timepoints (baseline, EOT and 1-month postpartum).

#### Depression

The Edinburgh Postnatal Depression Scale (EPDS (48)) was used to assess past-week depression symptoms. The EPDS is self-rated on a 4-point Likert scale with 10 items ranging from 0 to 3. A score of 13 or above indicates clinically significant depressive symptoms. The EPDS has shown to have adequate validity and reliability (56), and in our sample, reliability ranged from good to excellent (αs = .89 -.91).

#### Anxiety

The Generalized Anxiety Disorder-7 (GAD-7 (49)) assessed past 2-week anxiety symptoms. Seven items are rated on a 4-point scale, from 0 (*Not at all*), to 3 (*Nearly every day)*. Scores 5 or above indicate mild anxiety, 10 or above indicate moderate anxiety, and 15 or above indicate severe anxiety. The GAD-7 has demonstrated excellent validity and reliability in the general population (57) and in perinatal people (58). In our sample, reliability ranged from acceptable to good (αs = .74 -.89).

#### Physical activity

Physical activity was assessed via self-report and objective measurement. Participants completed the International Physical Activity Questionnaire-Short Form (IPAQ-SF (59)), a 7-item questionnaire about past 7-day activity, including minutes of vigorous activity, moderate activity, and walking. The IPAQ-SF has good psychometric properties (60). We examined how many days per week participants reported walking at least 10 minutes at baseline versus EOT. Participants’ activity was also assessed through Fitbit step counts, with average daily step counts compared at baseline and EOT.

Descriptive statistics are presented for participants’ demographic and reproductive characteristics, adverse events, compliance with attendance and Fitbit wear, patient satisfaction, physical activity levels, and cannabis use behaviors. Paired t-tests examined changes in clinical symptom levels (depression and anxiety) from baseline to EOT.

Participant responses during exit interviews were transcribed and analyzed using thematic analysis (61). One rater read all responses and developed a preliminary codebook of themes; themes were organized into topic areas related to the initial prompt and content of the responses: Once finalized, narrative responses were coded by both raters independently. In cases of disagreement, raters discussed discrepancies in coding to come to consensus. Ultimately, after all comments were coded, the final list of themes, and representative quotes, was developed.

## Results

### Participant characteristics

Participants were recruited from local OB-GYN practices and online advertisements. Brief telephone screenings were conducted with 221 individuals who expressed interest in the trial; of these 115 were deemed not eligible (primary reasons for lack of eligibility was being out of the gestation range or rates of cannabis use that were too low). Of the remaining individuals, 77 were screened and not interested or able to participate and 29 were scheduled for an in-person baseline assessment. Of 29 baselines scheduled, 16 people ultimately attended and were deemed eligible to enroll. As detailed in **Table 2**, enrolled participants (N = 16) had a mean age of 29.9 years (*SD* = 6.3). 62.5% of participants were White or Caucasian, 12.5% Black or African American, 18.8% biracial or multi-racial, and 6.3% endorsed other race. One quarter of the sample was Hispanic/Latina. Slightly over half (56.3%) were married, 18.8% were living with a committed partner, and 25% were single. Many participants (43.8%) had completed college, and some reported partial college (25%), high school/GED (25%), or a master’s degree (6.3%). Over half of participants (62.5%) were pregnant with their first child.

**Table 2:** Participant characteristics.

Baseline characteristic	*n*	*%*
Race		
Black or African American	2	12.5
White	10	62.5
Bi- or Multi-racial	3	18.8
Other	1	6.3
Ethnicity		
Hispanic/Latino	4	12.0
Not Hispanic/Latino	12	75.0
Marital status		
Single	4	25.0
Married	9	56.3
Living together with committed partner	3	18.8
Highest education level		
Completed high school/GED	4	25.0
Some college/Associate degree	4	25.0
Completed college/Bachelor’s degree	7	43.8
Completed Master’s degree	1	6.3
	*M*	*SD*
Age	29.88	6.28
Weeks gestation at baseline	19.73	3.76

### Feasibility, Safety & Acceptability of the Intervention

#### Feasibility

All individuals deemed eligible chose to participate in the WOW program and were cleared by their provider. Of 16 enrolled, 14 (88%) completed at least 4 of 6 sessions. Two participants had to discontinue participation due to medical reasons unrelated to the study (one after 2 weeks, the other after 4 weeks); among the remaining participants, the mean sessions attended was 5.8 out of 6. Though given the option to complete biweekly sessions in person or remotely, all participants elected to complete sessions remotely. Participants demonstrated strong compliance with Fitbit wear, with objective data showing that on average, participants wore trackers 10 or more hours per day on 95% of the days during the 10-week intervention period.

#### Safety

No participants reported adverse events related to study participation. Based on record review and self-report, there were no adverse infant outcomes. Among infants for whom we had complete birth record data (n=12), average gestation at time of delivery was 39.08 weeks (*SD* = 1.31), and average birth weight was 3.35 kilograms (*SD* = .37), or 7.39 pounds. No infants were born preterm; two babies (16%) had brief NICU admissions prior to discharge.

#### Acceptability

Participants reported high satisfaction with the intervention: mean CSQ-8 scores were 29.1 (*SD* = 4.9) out of a possible 32. Eleven participants participated in an optional exit interview. **Table 3** presents a list of the most common themes that arose. In brief, participants often noted that the intervention provided a sense of accountability that helped in achieving goals with regard to physical activity and PCU reduction. Many noted the structure of setting progressively higher goals, gradually, was useful in making behavior changes attainable. The Fitbit device was viewed as easy to use and helpful in achieving step-count goals. Participants noted that barriers (e.g., poor weather, low energy) would sometimes get in the way of increasing activity. Regarding physical activity as influencing mood or cannabis cravings, some individuals stated that they did not believe increased activity altered mood or cravings, while others noted that activity changes did lead to mood improvements or helped manage cravings. Some remarked on the interconnection between mood and cravings, observing that increased activity would at times improve mood, thus diminish cravings for cannabis.

**Table 3:** Participant feedback regarding women out walking intervention.

Theme	Definition	Representative quotes
Intervention Provided Accountability	Intervention helped provide participants with accountability for their physical activity and cannabis use goals	*“Knowing that I had a call with somebody every two weeks to talk about where I was…. Knowing that I was going to be held accountable for at least talking about why I didn’t make a goal…. I had that in the back of my head.”* *“Checking in … about me decreasing my usage, because again that was my goal to decrease or to stop. That was really helpful. [I] didn’t feel questioned about [using cannabis] or anything.It actually felt like a helpful and supportive* sp*ace to get accountability for that goal”* *‘It helped me stay motivated … I knew I had something that would hold me accountable.’*
Gradual Step-count Increases Helpful	Setting gradual increases in physical activity goals provided structure to change behavior in an achievable way	*‘And that, for me, was very positive … I liked the [physical activity] goals that were set, because they were attainable."* *“[The intervention offered] a more supportive way to get to goals. And, not reaching a goal wasn’t like a “damned if you do, damned if you don’t” situation”*
Fitbit Device was Easy to Use	The Fitbit device was easy to use. Tracking steps was helpful in achieving goals	*“I definitely liked the monitoring of the Fitbit and that it connected to the phone … I'm not a tech savvy person, but that was really easy to use.”* *“I started wearing the watch. It motivated me more to accomplish step goals everyday”* *“And just having the [Fitbit] and checking in every day and saying, “Oh alright, I’m at 5400. If I walk to the mailbox, I’ll get to 7000. Cool.” I’m calculating distances like that.”*
Barriers to Physical Activity would Arise	Barriers would sometimes get in the way of increasing physical activity levels	*“I had a lot of medical appointments during my pregnancy. So that was a barrier.”* • *“[Barriers were] mainly weather-wise. The switch into colder time of winter. Started snowing, getting icy.”*

### Preliminary efficacy

This trial was not designed to test intervention efficacy; however we examined changes over time in a few key variables (cannabis use, depression, anxiety, physical activity) to yield a preliminary indication of the intervention’s potential.

#### Cannabis use

**Figure 1:**
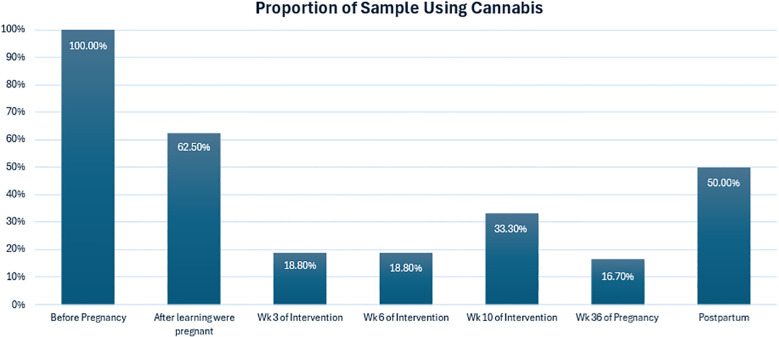
Proportion of sample using cannabis.

On the TLFB, all participants (100%) reported using cannabis regularly (2+ times per week) in the 90 days prior to pregnancy. Upon learning of pregnancy, 6 of 16 participants (37.5%) discontinued use; thus, 10 of 16 participants (62.5% of the sample) were using cannabis at the start of the intervention. Rates of cannabis decreased during the intervention period (**Figure 1**). By 36 weeks gestation, only 2 of 12 participants completing that assessment (16.6% of the sample) were using cannabis. After giving birth, more participants had returned to use: 5 of 10 participants (50%) were using cannabis at the 1-month postpartum timepoint. In comparing self-reported cannabis use on the TLFB and urine toxicology results (collected during in person time points), there were no cases in which cannabis use was denied on the TLFB, yet had a positive test result on the toxicology screening.

During pregnancy, among those who continued to use cannabis, women used cannabis an average of 41% of days preceding week 3 (n=3; range from 10% to 97% of days), 40% of the days preceding week 6 (n=3; range from 4.8% to 100% of days), and 45% of days preceding week 10 (n=4; range from 2.9% to 100% of days). Of the small subset of women who were still using cannabis at 36 weeks gestation (n = 2), the average number of days of use was 58% (range from 15.8% to 100% of days). At postpartum, when more participants had returned to cannabis use, the average number of days using was 13% (n=5; range from 1.7% to 32% of days).

#### Depression and anxiety

**Figure 2:**
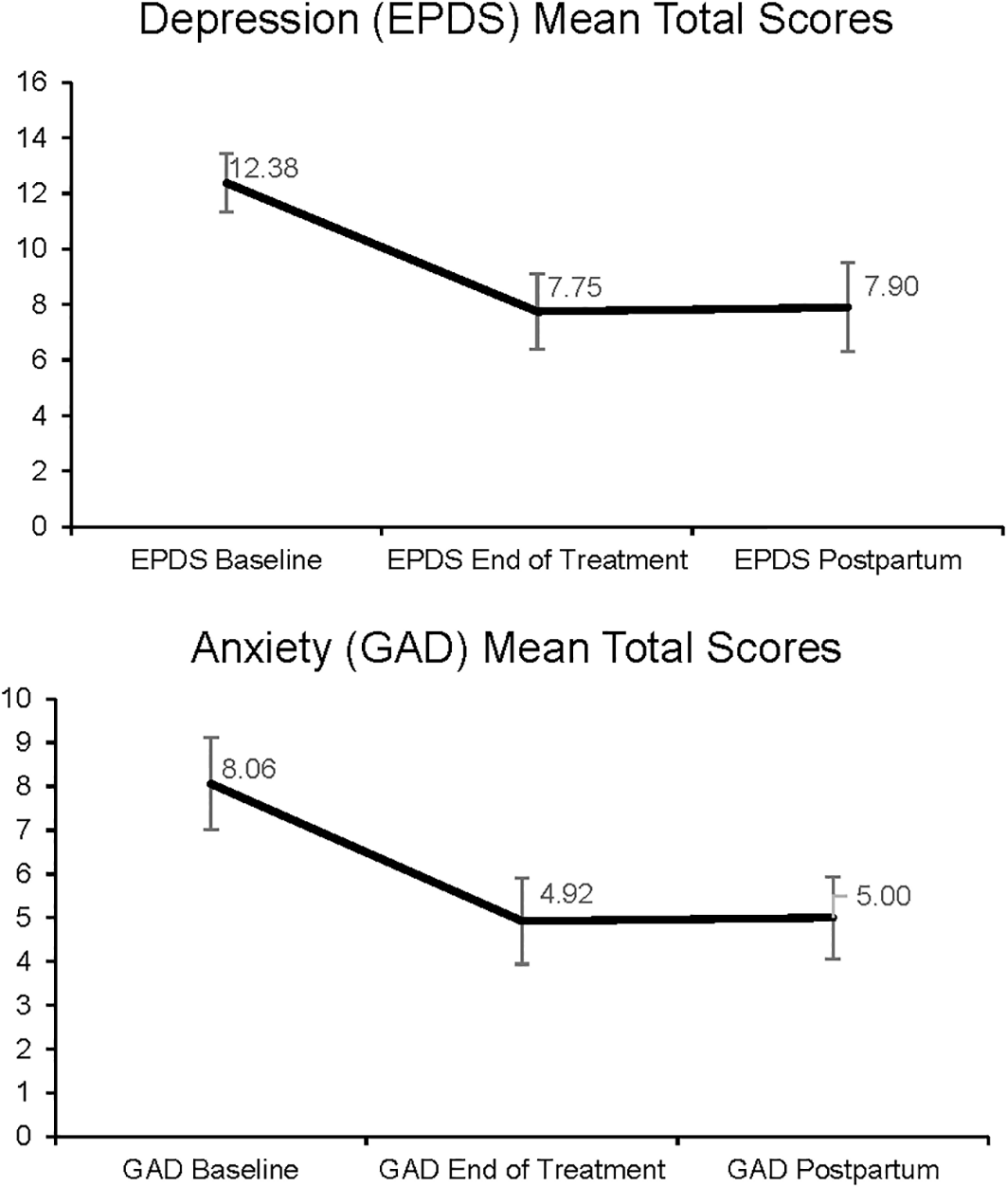
Changes in depression and anxiety symptoms.

Paired sample t-tests indicated that average depression (EPDS) scores decreased significantly from baseline (*M* = 12.91, *SD* = 3.30) to end of treatment (*M* = 7.91, *SD* = 4.89), *t*[10] = 4.28, *p = .*002, *d* = 1.35). Similarly, mean anxiety (GAD-7) scores decreased significantly from baseline (*M* = 8.27, *SD* = 4.54) to end of treatment (*M* = 5.18, *SD* = 3.43), *t*[10] = 2.45, *p = .*03, *d* = 0.77). As shown in **Figure 2**, reductions in depression and anxiety were maintained across the intervention and through postpartum.

#### Physical activity

Based on objective measurement through Fitbit wear, participants’ average daily step count was 5738 steps/day (SD = 1554) at baseline, prior to the WOW intervention. At end of treatment, participants average daily step count was 6562 steps/day (SD = 2023) based on the 7-day period prior to assessment. Additionally, based on women’s self-report of physical activity on the IPAQ-SF, differences were observed over the course of the intervention: at baseline, 35.7% of the sample reported walking four or more days a week, whereas at EOT, 63.6% reported walking four or more days a week.

## Discussion

The goal of the current study was to examine the feasibility, acceptability, safety, and preliminary efficacy of Women Out Walking, a physical activity intervention designed for pregnant women seeking to reduce cannabis use. We observed strong compliance in session attendance as well as wearing the activity monitor. Participants voiced a high satisfaction with the intervention, often noting that it was useful in providing a sense of accountability and structure in pursuing their cannabis use and physical activity goals. Participants did not report any adverse events related to the study intervention. Rates of cannabis use among participants were lower by end of intervention in terms of percent days of use during each reporting period. Clinically meaningful decreases were measured in participants’ symptoms of depression and anxiety. Levels of physical activity increased slightly over the course of the intervention, both by self-report and objectively-determined physical activity levels. Although participants’ physical activity behavior did not increase dramatically across the intervention period, these increases in daily activity are notable given that participants were at an advanced gestation of pregnancy at EOT, when it is typical for activity levels to decrease (62). Although preliminary, results of this pilot trial are promising, suggesting that pregnant cannabis users who wish to discontinue or decrease PCU may be able to engage with this type of intervention.

Given that public health guidelines advise discontinuation of cannabis during pregnancy, and yet few interventions exist to help individuals accomplish this, a tailored physical activity intervention could represent one strategy for supporting pregnant patients who seek to quit or reduce PCU. The benefits of a physical activity approach may not only include reduction in use, but also broader benefits to health and mood. Given that many people who continue to use cannabis while pregnant endorse motivations for use that involve addressing pregnancy symptoms (e.g. appetite disturbance, nausea) or psychological distress (e.g. stress, anxiety (63)), it is valuable to consider whether physical activity - particularly bouts of “in the moment” activity - may help curb cravings and lower PCU by improving symptoms that may prompt use. Indeed, several participants remarked that a benefit of the intervention was that physical activity helped improve their mood, and decreased distress helped them avoid PCU.

A number of participants remarked that the sessions with a clinician were motivating, providing accountability and support. This is helpful feedback to incorporate in the development of future physical activity interventions, particularly as some approaches to promoting increased activity levels do not involve interventionist contact. Sessions with an interventionist increase cost, however they may provide an essential feature for some individuals to stay engaged and change behavior. Future research could evaluate the best setting in which to provide this type of intervention, keeping in mind that some pregnant people may be reluctant to speak about cannabis use behavior in medical settings (32).

Not surprisingly, some participants noted barriers to increasing physical activity during pregnancy. It will be useful to consider how future versions of this intervention can address barriers that are typically encountered (e.g., inclement weather, childcare). While study interventionists provided suggestions to overcome such barriers, additional strategies may be needed help pregnant individuals facing significant logistical obstacles. This intervention was designed for those seeking to reduce or abstain from PCU; however, it is also important to consider how this type of intervention could be modified for related populations, such as those who are at risk for returning to cannabis use in the postpartum period. Normal post-birth issues such as physical pain and discomfort, sleeping difficulties, and the stress of newborn care could elevate risk of use, even among those who intend to remain abstinent after the baby’s birth.

Should physical activity prove to be efficacious in lowering rates of PCU, it would be worthwhile to examine mechanisms that may account for this effect. Prior research suggests that moderate aerobic exercise may activate the receptors within the endocannabinoid system (64, 65). Although research on neurobiological effects of exercise on the endocannabinoid system is preliminary, the idea that the effects of exercise might mimic effects of cannabis suggests that exercise may be especially effective in reducing cannabis use (66). Research with non-perinatal populations have found that greater physical activity is associated with lower levels of cannabis use and greater success reducing use: patients who expressed interest in a self-guided cannabis quit attempt who were more physically active were more likely to be successful than less active participants (67). In another study, non-treatment-seeking people with cannabis use disorder who completed ten, 30-minute exercise sessions over two weeks reported lower cravings for cannabis and lower use during and following the exercise period (68). Although these studies were not randomized controlled trials, they provide support for the potential value of physical activity as a tool to reduce cannabis use.

This study is the first to examine a physical activity program as an intervention to promote reduction of cannabis use during pregnancy. The assessment of PCU by trained interviewers, with repeat assessments over time, was a strength in the design. Another strength was use of objective measurement of physical activity across the intervention period to corroborate self-reports. As a nonrandomized pilot trial, this study was designed specifically to examine the feasibility and acceptability of the intervention, not to evaluate intervention efficacy. Nonetheless, the preliminary findings we obtained regarding participants’ reductions in PCU, increases in physical activity, and improvements in depression and anxiety are promising. These outcomes require rigorous testing in a fully-powered randomized controlled trial. In the next phase of our research, which is now underway, the WOW intervention will be compared to a Fitbit only condition, which will be important to determine the relative importance of the interventionist sessions with feedback and support, in addition to the potential impact of Fitbit wear only.

Given the sharp rise in cannabis use in pregnant populations, it is critical to develop strategies to support individuals in lowering their use of cannabis during pregnancy. We found strong support for the feasibility, acceptability, and safety of a physical activity intervention to help women reduce prenatal cannabis use. Though preliminary, indications suggest that the intervention may be effective in reducing PCU, as well as promoting other positive outcomes. A fully-powered randomized trial is required to more definitely assess efficacy of the WOW intervention. In addition, future research is needed to identify the optimal ways to implement interventions addressing PCU within the real world of clinical care, including strategies to improve patient-provider communications about substance use in pregnancy.

## Data Availability

The raw data supporting the conclusions of this article will be made available by the authors, without undue reservation.
